# Cost-Sensitive Classification for Evolving Data Streams with Concept Drift and Class Imbalance

**DOI:** 10.1155/2021/8813806

**Published:** 2021-08-02

**Authors:** Yange Sun, Meng Li, Lei Li, Han Shao, Yi Sun

**Affiliations:** ^1^School of Computer and Information Technology, Xinyang Normal University, Xinyang 464000, China; ^2^Henan Key Lab of Analysis and Applications of Education Big Data, Xinyang Normal University, Xinyang, China; ^3^Institute of Zhengzhou Information Science and Technology, Zhengzhou, China

## Abstract

Class imbalance and concept drift are two primary principles that exist concurrently in data stream classification. Although the two issues have drawn enough attention separately, the joint treatment largely remains unexplored. Moreover, the class imbalance issue is further complicated if data streams with concept drift. A novel Cost-Sensitive based Data Stream (CSDS) classification is introduced to overcome the two issues simultaneously. The CSDS considers cost information during the procedures of data preprocessing and classification. During the data preprocessing, a cost-sensitive learning strategy is introduced into the ReliefF algorithm for alleviating the class imbalance at the data level. In the classification process, a cost-sensitive weighting schema is devised to enhance the overall performance of the ensemble. Besides, a change detection mechanism is embedded in our algorithm, which guarantees that an ensemble can capture and react to drift promptly. Experimental results validate that our method can obtain better classification results under different imbalanced concept drifting data stream scenarios.

## 1. Introduction

Data stream classification has attracted much attention in the scenario of big data mining due to its presence in many real-world fields, such as social network analysis, weather prediction, online medical diagnosis, and weblog mining [1–5]. Concept drift is a common feature of data streams [6–9], which refers to the phenomenon of target concepts of streams changing over time. Concept drift can deteriorate the performance of classiﬁcation because the model trained on old concepts may be unsuitable for new concepts. For example, fashion trends in recommend systems may be influenced by customer behavior, and the weather forecast model may no longer be applicable as the season changes. Therefore, an efficient data stream learning model should have the capability of capturing drifts promptly and updating the model accordingly [7, 10].

A growing number of methodologies have been proposed for dealing with concept drift [9]. Among these techniques, the window-based method adopts a natural way of forgetting mechanism to add new instances and eliminate outdated instances. The sliding window is the most frequently used window technology. It adopts the first-in-first-out structure to move on processed instances and ensure that the current window stores the latest instances. Because ensemble algorithms have the advantage of modularity and can quickly adapt to changes, ensemble-based methods are the most common methods for handling concept drift.

Although much work has been done on concept drift [5–7], the class imbalance problem [11] (i.e., negative class instances are more extensive than other classes) further increases the difficulty of addressing concept drift [12]. Class imbalance commonly exists in the real world. Examples include cancer diagnosis, financial fraud detection, and geological disaster prediction. For binary classification, the class that has more instances is called the majority class (negative class), and the other is the minority class (positive class). For example, in the online fraud identification of automobile insurance, fraudulent customers accounted for only 1% of the total customers in 100 000 instances. Finding a way to identify only 1% of fraudulent instances correctly can significantly reduce economic loss.

Several popular methods for dealing with the class imbalance issue [13–18] can be broken down into main groups: data-level techniques, cost-sensitive learning, and ensemble methods. Cost-sensitive learning methods aim to minimize the total cost. Some researchers argue that the cost-sensitive strategy is the most effective and frequent technique for dealing with class imbalance [11].

How to tailor the cost-sensitive learning strategy and adapt it to a nonstationary environment to enhance the capability of dealing with class imbalance is meaningful work. In practice, constructing classifiers under evolving data streams existing class imbalance is not a trivial task. It should address the following subproblems: (1) How can concept drift be handled? (2) How can class imbalance be managed?

A novel cost-sensitive learning scheme, named Cost-Sensitive based Data Stream (CSDS), is devised to tackle the combined issue to address these challenges. The contributions are threefold:

1. A novel cost-sensitive variant of the ReliefF algorithm, named Cost-Sensitive based on ReliefF (CS-ReliefF), is proposed. The CS-ReliefF considers cost information in feature weighting to address the class imbalance issue at the data level.
2. A dynamic cost-sensitive weighting mechanism is developed in the classification stage, incorporating cost value into the learning to alleviate the class imbalance at the algorithm level.
3. The performance of our algorithm was implemented on different kinds of class imbalance data stream benchmarks. The results demonstrated that CSDS achieves the best overall performance in *G*-mean, running time, and concept drifts adaption.

## 2. Related Work

### 2.1. Class Imbalance Learning for Static Data

Researchers have done several works on class imbalance classification on static datasets [11]. The research work is mainly divided into three categories: data preprocessing techniques, cost-sensitive learning methods, and ensemble-based methods [13].

Data preprocessing techniques are mainly to alleviate the influence of class imbalance employing changing the original data distribution. Undersampling and oversampling are two common data preprocessing technologies. The undersampling method balances the classes by deleting the majority of instances, resulting in information loss [14]. The oversampling technique balances the data by duplicating a minority of instances. However, due to the uncertainty in the synthesis of new instances, it may weaken the classifier's performance [15]. SMOTE [16] is the most famous random oversampling algorithm, synthesizing new minority instances near the original minority instances. However, it often results in overfitting.

Cost-sensitive learning solutions [17] assign different costs to different classes, which seek to minimize the total cost. Suppose that the majority class is misclassified as a minority class. In that case, a lower misclassification cost is assigned, and when a minority class is misjudged as a majority class, a higher misclassification cost is assigned. In this way, we could balance the class distribution of the data. Most of these methods extend traditional machine learning methods to make them cost-sensitive. For example, literature [18] introduced cost-sensitive strategies into the SVM algorithm to minimize cost-sensitive hinge losses. AdaCost algorithm proposed in literature [19] reduces the weight of misclassified instances by introducing a cost-sensitive weight function into the AdaBoost. Sun et al. presented a series of algorithms based on cost-sensitive learning [20].

Bagging and boosting are two commonly used strategies in ensemble algorithms. Representative bagging-based ensemble algorithms used to deal with class imbalance include OverBagging [21], UnderBagging [22], UnderOverBagging [23], and DES-MI [24]. Data preprocessing techniques are often used in boost-based algorithms. Representative methods include SMOTEBoost [25] and RUSBoost [26]. Some ensemble methods combine both bagging and boosting strategies, including EasyEnsemble and BalanceCascade [27].

### 2.2. Data Streams Learning under Concept Drift and Class Imbalance

Although many efforts have been made focusing on class imbalance or concept drift separately [28–32], the combination of the two issues in data stream classification has not yet drawn enough attention.

Gao et al. proposed a general framework, called Sample and Ensemble (SE), for addressing class imbalance issues under streaming scenarios [28]. The SE divides the continuously arriving block into two groups: majority class instances and minority class instances. And then, SE collects the minority instances of the previous blocks and removes some of the majority class instances from the current chunk. Chen and He [29] introduced a novel ensemble solution, called Recursive Ensemble Approach (REA), for tackling class imbalance issues under a nonstationary environment. REA utilized the *K*-NN algorithm to measure the similarity between the minority class instances of the previous block and the minority class instances of the current block and chose the previous minority instances to balance the classes of the current block. Polikar et al. [30] presented an algorithm based on the Learn^++^ framework [31] to deal with class imbalance under a data stream environment named Learn^++^.NIE. Recently, Mirza et al. introduced an online version of Extreme Learning Machine to solve the class imbalance issue [32].

In [33], a novel neural networks framework based on a cost-sensitive strategy was devised for handling the class imbalance issue. Li et al. introduce an ensemble algorithm using a multiwindow strategy to handle class imbalance issues [34]. More specially, three windows are designed in the algorithm: the current data block, the latest minority instances, and the pool of base classifiers. Lu et al. extended and improved the classic dynamic weighted majority (DWM) to effectively deal with the imbalance issue and named Dynamic Weighted Majority for Imbalance Learning (DWMIL) [35]. Moreover, DWMIL used an underbagging strategy during data preprocessing to handle class imbalance. However, it has the drawback of overfitting. Zyblewski et al. proposed a dynamic classifier ensemble selection for imbalanced drifted data streams [36]. Most recently, Cano and Krawczyk proposed an algorithm called Kappa Update Ensemble (KUE) [37], which utilized the Kappa statistic for dynamically updating weights of base classifiers.

Simultaneously, some common problems exist in imbalanced data stream classification methods: these algorithms can deal with a specific type of concept drift. Besides, class imbalance often exists in the data stream together with concept drift. Most algorithms only focus on one problem and do not fully consider two issues simultaneously.

## 3. Our Method

### 3.1. Cost-Sensitive Based Data Stream Algorithm

**Figure 1:**
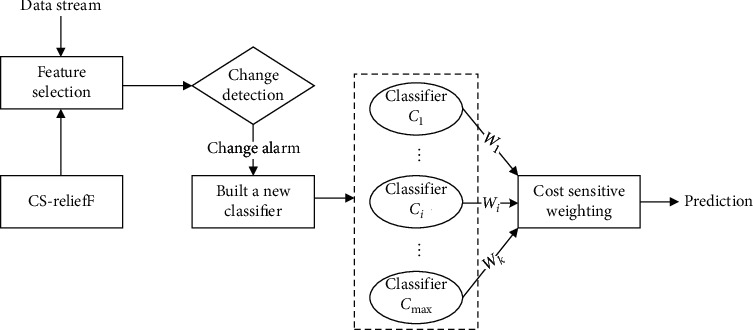
The framework of CSDS.

A novel ensemble framework based on cost-sensitive feature selection is introduced to handle this study's joint issue. As shown in Figure 1, the proposed algorithm primarily consists of four steps:

  Step 1: Data preprocessing: a cost-sensitive feature selection based on the ReliefF algorithm, named cost-sensitive ReliefF (CS-ReliefF), is devised. CS-ReliefF incorporates the cost information into feature selection, which selects a subset of features helpful in identifying the minority class. Hence, the feature set is more meaningful for effective prediction and has the effect of dimension reduction.
  Step 2: Change detection: our algorithm employs concept detection to capture the changes explicitly, and when concept drift is detected, a new member classifier is built on the latest data.
  Step 3: Classification module: a novel weighting scheme, that is, the weight of the base classifier, is updated based on accuracy and the total cost of misclassification on the latest data.
  Step 4: Prediction: the weighted majority voting rule is used for predicting unknown instances.

### 3.2. Cost-Sensitive ReliefF Algorithm

A novel cost-based feature selection, named Cost-Sensitive ReliefF algorithm (CS-ReliefF), is proposed in this section. We adopt the ReliefF algorithm [38] mainly because it is simple, fast, and effective. More specially, we tailed the famous feature select algorithm ReliefF into a cost-sensitive learning model, which takes advantage of cost information into account during feature selection.

The main idea of the ReliefF algorithm is to weigh features according to their classification contribution. Specifically, the ReliefF randomly selects an instance *x*_*i*_ with class value *y*, finds its *k* nearest neighbors from the same class and different classes, and is denoted by *H*_*j*_ and *M*_*j*_ (*y*), respectively. It updates the weights of all features based on their ability to distinguish neighboring instances.

Let *x*_*i*_ and *x*_*j*_ denote two instances, and their classes are *y*_*i*_ and *y*_*j*_. The function diff *(f*, *x*_i_, *x*_*j*_) is defined as the difference between the value of feature *f* for two instances *x*_*i*_ and *x*_*j*_, and it can be calculated according to

(1) $$\begin{array}{c} \text{diff}\left(f,x_{i},x_{j}\right)=\left\{\begin{array}{cc} 0, & y_{i}=y_{j},\\ 1, & y_{i}\neq y_{j}. \end{array}\right) \end{array}$$

If the class is numerical, *i*.e., *y* ∈ *R*

(2) $$\begin{array}{c} \text{diff}\left(f,x_{i},x_{j}\right)=\left|\frac{y_{i}-y_{j}}{\max_{f}-\min_{f}}\right|, \end{array}$$

where max_*f*_ and min_*f*_ represent the maximum and minimum values of *f*, respectively, and the diff *(f*, *x*_i_, *x*_*j*_) reflects the discrimination between *x*_i_ and *x*_*j*_ on *f*.

Let *W*_*f*_ denote the influence of feature *f*, where *W*_*f*_ ∈ [−1, 1]. ReliefF initializes the weights of all features to zero firstly. Then, the RelifF randomly selects an instance *x*_*i*_ and searches its *k* nearest neighbors. The ReliefF updates the weight of each feature according to

(3) $$\begin{array}{c} W_{f}=W_{f}-\frac{\sum_{j=1}^{k}\text{diff}\left(f,x_{i},H_{j}\right)}{r\cdot k}+\frac{\sum_{y\neq y_{i}}\left[\left(P\left(y\right)/1-P\left(y_{i}\right)\right)\sum_{j=1}^{k}\text{diff}\left(f,x_{i},M_{j}\left(y\right)\right)\right]}{r\cdot k}, \end{array}$$

where *P* (*y*) is the prior probability of class *y* estimated from the training set, and *r* is a user-defined parameter indicating the number of iterations.

Unlike ReliefF, the proposed CS-ReliefF algorithm updates *W*_*f*_ considering cost information according to equation (4). In this way, the CS-ReliefF algorithm tends to select features with low costs.

(4) $$\begin{array}{c} W_{f}=W_{f}-\frac{\sum_{j=1}^{k}\text{diff}\left(f,x_{i},H_{j}\right)}{r\cdot k}+\frac{\underset{y\neq y_{i}}{\sum }\left[\left(P\left(y\right)/1-P\left(y_{i}\right)\right)\sum_{j=1}^{k}\text{diff}\left(f,x_{i},M_{j}\left(y\right)\right)\right]}{r\cdot k}-\lambda \frac{{\text{Cost}}_{f}}{r\cdot k}, \end{array}$$

where *Cost*_*f*_ is the test cost of *f*, and *λ* is the influence factor specified by the user.

*Cost*_*f*_ is generated by a normal distribution, and the cost function is defined as follows:

(5) $$\begin{array}{c} f\left(x\right)=\frac{1}{\sqrt{2\pi \sigma^{2}}}e^{\left({\left(x-\mu \right)}^{2}/\sigma^{2}\right)}, \end{array}$$

where *μ* and *σ*^2^ are the mean and variance. To avoid the randomness of one sampling, the above process needs to be iterated *r* times. In our algorithm, the parameter *p*% is selected to adapt to the dynamically changing feature space. We adopt *p* as 75 in the following experiments. The pseudocode is shown in Algorithm 1.

### 3.3. Cost-Sensitive Ensemble Learning Method

Let *E* = {*C*_1_, *C*_2_,…, *C*_*k*_} represent an ensemble with *k* base classifiers. The CSDS uses a sliding window technique to divide data stream *S* into data blocks *B*_*i*_, *i* = 1, 2,…, *n*, where |*B*_*i*_| = |*B*_*j*_| = *d*, *i* ≠ *j*. More specially, whenever a new instance at time *j* is observed and inserted into the window, the *j* − |*W*| is discarded. When a new block *B*_*i*_ arrives or a change occurs, we evaluate the effect of features according to equation (4) and use this to select an effective feature subset *F'* ⊆ *F* (refer to Section 3.2). The CSDS uses *B*_*i*_ to build a new classifier *C′* and weights it according to the following equation:

(6) $$\begin{array}{c} W\left(C^{\prime }\right)=\frac{1}{{\text{MSE}}_{r}+\alpha }. \end{array}$$

The CSDS weights each base classifier *C*_*i*_ ∈ *E* according to the following equation:

(7) $$\begin{array}{c} W\left(C_{i}\right)=\frac{1}{{\text{Cost}}_{i}+{\text{MSE}}_{r}+{\text{MSE}}_{i}+\varepsilon },\;i=1,2,\ldots ,k, \end{array}$$

where MSE_*r*_ represents a randomly predicting classifier's performance of, and is employed as, the baseline for predicting the current distribution; MSE_*i*_ represents the mean square error of *C*_*i*_ on *B*_*i*_ at time *t*, respectively; *Cost*_*i*_ is the total misclassification cost of *C*_*i*_ on *B*_*i*_. When the ensemble is full, the worst classifier is removed, and the newly learned classifier is added to the ensemble. Our method adopts the weighted voting rule to make the final prediction. Moreover, the proposed algorithm utilizes the Drift Detection Method (DDM) [39], a change detection schema by detecting the classification model's error rate, as the change detector. In fact, it could be any change detection algorithm. The pseudocode of the cost-sensitive-based data stream algorithm is shown as follows (Algorithm 2).

## 4. Experiments and Analysis

The experiments were carried out on the Scikit-Multiflow framework [40], which is a python platform based on popular open-source frameworks including scikit-learn and MOA [41]. It includes data stream generators, classification algorithms, and evaluation methods.

### 4.1. Data Benchmarks

In the following experiments, we employed eight data stream benchmarks, including synthetic and real-world streams. The stream generators generated the synthetic streams in the Scikit-Multiflow framework. We adopted the ConceptDriftStream generator to simulate concept drift and used the ImbalancedStream generator to set the class imbalance rate (^#^majority instances: ^#^minority instances). The description of the streams is shown in Table 1.

The Hyperplane that simulates a *d*-dimensional hyperplane is the most popular synthetic data to simulate gradual concept drift. A gradual drift stream with 1 m instances was generated in our experiments, and its imbalance rate was set to 5.

The SEA dataset is the most commonly used dataset representing sudden drift scenarios in data stream mining. We use the data stream generator to generate a data set of a sudden change in concept recurrence. The data set has a total of 1 m instances, which reappear every 250 K instances. Each instance is described by three attributes, which are used to represent one of the four concepts.

The LED dataset contains data used to predict the seven-segment LED display. We chose the 24 attributes version of the LED. We generate a mixed drifts stream containing 1 m instances, including sudden and gradual concept drifts.

The Rotating spiral is a dataset with the class imbalance and gradual concept drift. It is used to describe four types of spirals. The rotating spiral data stream contains 1 m instances, and the imbalance rate is 19.

The Spam dataset is a representative imbalanced real-world dataset, which collects e-mail messages from the Spam Assassin Collection. It has 9324 instances with 500 attributes. There are two classes (legitimate and spam). The imbalance rate of spam is 4. We simulate the spam into a stream with gradual drift by changing the features of spam over time.

The Sensor dataset has 2219803 instances, and five attributes describe each instance. The data is the information of 54 sensors of Intel Berkeley Research Lab in two months. Since attributes such as brightness and temperature change over time, the stream may contain concept drift.

The Electricity dataset is one of the most widely used real-world datasets. It was collected from the Electricity Market in Australia, containing 45312 instances, each described by seven attributes. The purpose of this dataset is to predict whether the price of electricity will increase (up) or decrease (down) with changes in market demand and supply. The classes are approximately balanced.

The Airlines stream contains 539383 instances, and eight attributes describe each instance. The class of Airlines is a delay, which indicates whether the flight is delayed.

### 4.2. Evaluation Metrics for Class Imbalance Learning

The *G*-mean is the geometric means of the recall of abnormal classes and that of normal classes, often used to measure the classifier's ability to handle unbalanced data [11]. It is often applied in data streams with class imbalance to reduce the bias of the overall accuracy. For binary class classification, the *G*-mean is as follows [42, 43].

(8) $$\begin{array}{c} G-\text{mean}=\sqrt{\frac{\text{TP}}{\text{TP}+\text{FN}}\times \frac{\text{TN}}{\text{TN}+\text{FP}}}. \end{array}$$

*G*-mean can be extended to multiclass cases. Assuming that there are *m* classes, *G*-mean is still the geometric average of various correct rates, defined as

(9) $$\begin{array}{c} G-\text{mean}={\left(\prod_{i=1}^{m}G-{\text{mean}}_{i}\right)}^{1/m}, \end{array}$$

where G-mean_*i*_ is *G*-mean of *i*th class.

### 4.3. Experimental Results

We verified the effectiveness of CSDS using cost-sensitive strategies in evolving data stream scenarios involving different types of drifts and class imbalance. CSDS was compared with the following methods:

- VFDT: VFDT is an incremental decision tree classification based on the Hoeffding inequality theory, which can guarantee the constructed decision tree's accuracy with a certain probability.
- AUE2: AUE2 is a block-based ensemble that combines the accuracy-based weighting mechanism with the incremental learning of the Hoeffding tree and aims to deal with various types of drift.
- KUE: KUE is a dynamic weighting ensemble that utilized the Kappa statistic to update base classifiers' weight dynamically.

The evaluation can generate an incremental learning curve of metrics changing over time. For a fair comparison, the maximum number of the compared ensemble algorithms was set to 15. We chose the Hoeffding tree as the base classifier. The performance can be evaluated concerning *G*-mean and time (the averaged results of 10 runs), as shown in Tables 2 and 3.

#### 4.3.1. *G*-Mean Analysis

Concerning the *G*-mean, CSDS achieved the best average ranking, as shown in Table 2. CSDS gained the best performance over four data streams: HyperPlane, SEA, Spam, and Electricity. We find that CSDS classification performs better in class imbalance data streams environment. VFDT obtains the worst performance. This is because it cannot solve the class imbalance challenge, but also incapable of dealing with concept drift. CSDS classification employs the cost-sensitive learning strategy during the data preprocessing and classification stages. CSDS uses the CS-ReliefF algorithm to incorporate cost information into feature selection to select a subset of features helpful in identifying minority classes. Therefore, the feature set is more meaningful for effective prediction and has the effect of dimension reduction. Simultaneously, a dynamic cost-sensitive weighting strategy is developed to reduce class imbalance at the algorithm level.

#### 4.3.2. Time Analysis

In terms of running time, VFDT performs best, followed by our algorithm, and KUE performed the worst. As shown in Table 3, we observe that the ensemble algorithms have certain advantages in *G*-mean, but it does not perform well in running time. Although the single decision tree classifier VFDT has apparent advantages in time, it performs the worst in overall performance. Overall, in most cases, CSDS can achieve a good compromise between *G*-mean and running time and adapt to drifts faster than other ensemble methods. Our algorithm benefits from the modular characteristics of ensemble learning, which can better deal with recurring gradual drifts. Meanwhile, the change detection mechanism is embedded in our algorithm to capture sudden drift in time.

Next, we adopt graphical plots to visualize how the algorithm is affected by different kinds of change. *X*-axis and *y*-axis denote the number of processed instances and *G*-mean of the algorithms, respectively.

**Figure 2:**
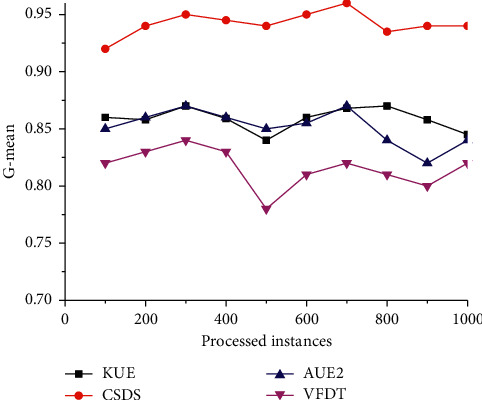
Comparison of *G*-mean on the SEA dataset.

The SEA dataset is used to simulate scenes with sudden changes and to detect the ability to address sudden concept drift. In this scenario, the curves of the *G*-mean with the increment of processed increases are shown in Figure 2. The performance of the VFDT is the worst, followed by AUE2 and KUE, and CSDS is the best. Moreover, around the 50Kth instance, the *G*-mean values of all algorithms undergo rapid fluctuations except CSDS. This may benefit from the concept drift detection mechanism, which can promptly capture a sudden drift, thereby establishing a new classifier to adapt such drift.

**Figure 3:**
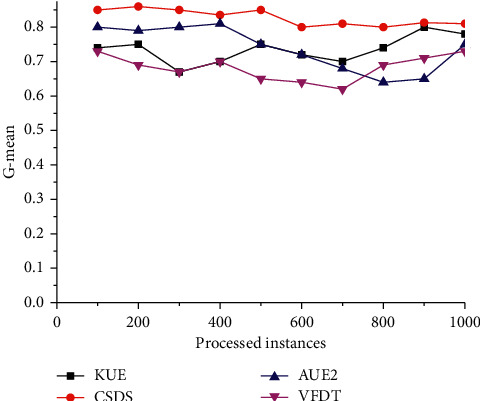
Comparison of *G*-mean on rotating spiral dataset.

The LED dataset simulates mixed concept drift, that is, scenes with gradual and sudden concept drift. It is intended to verify the algorithm's responsiveness to mixed drift. Specifically, the dataset is a stream containing two gradual drifts and one abrupt concept drift. When processing half of the stream, the target concept suddenly shifts from one concept to another. As shown in Figure 3, we have observed that all algorithms maintain a higher *G*-mean value when the data is relatively stable. When a sudden change occurs in the data stream, the performance of all algorithms except CSDS drops sharply. This may be because CSDS can capture different kinds of changes timely and reconstruct a new model to recover from concept drift quickly. Besides, CSDS provides the best overall performance utilizing cost-sensitive learning strategies in feature selection and classification.

**Figure 4:**
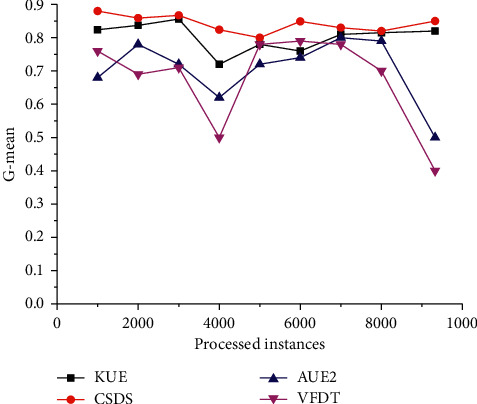
Comparison of *G*-mean on spam dataset.

Changes in real-world streaming scenarios are often complex and changeable, so simulating the real-world environment can better verify classifiers' performance.  Figure 4 illustrates the *G*-mean curves on the Spam data stream change over time. All the curves show varying degrees of fluctuation, which implies that there may exist a drift in the stream. Since VFDT and AUE2 cannot deal with imbalances, the dataset simulates a real-world nonstationary scenario that includes class imbalance. They perform poorly in a scenario where class imbalance and concept drift coexist.

In contrast, the curves of CSDS and KUE are not significantly affected by concept drift, since CSDS is oriented to the data stream's changing characteristics to respond to these problems quickly and in real-time. Additionally, cost-sensitive learning strategies are adopted at the data level (feature selection) and algorithm level (classifier weighting) to effectively deal with imbalances.

**Figure 5:**
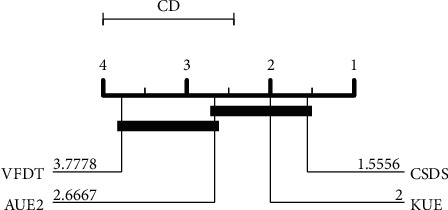
Nemenyi test for *G*-mean of all algorithms.

Finally, we adopted the nonparametric Friedman test with a significance level *α* = 0.05 to perform statistical tests on all competitive algorithms [44]. The statistical test results show that the null hypothesis is rejected. That is, there is no significant difference between the algorithms. After that, we further employ the Nemenyi test [45] to verify whether the performance of our method is statistically different from other algorithms. The result is shown in Figure 5. The results show that CSDS is significantly better than VFDT.

## 5. Conclusion

This study provides novel insight into how to utilize a cost-sensitive learning strategy to deal with class imbalance under dynamic streaming scenarios. An ensemble schema based on a cost-sensitive strategy is devised to handle the combination of the two issues. Firstly, a cost-sensitive version of the ReliefF algorithm that incorporates cost information during the data preprocessing is proposed to solve the class imbalance issue at the data level. Secondly, a cost-sensitive classifier weighting scheme utilizing cost information is devised in the ensemble stage. Moreover, a change detection module is embedded in the ensemble to capture drift in real-time. Finally, extensive experimental results show that our method is superior to the competitive algorithms and gains the best trade-off between performance and resources, especially for nonstationary data streams with imbalanced class environments. Furthermore, the results verified its statistical significance with a nonparametric Friedman test.

This study focuses on the topic of single-label data stream classification. Multilabel data streams are common in many real applications. In the future, we plan to extend cost-sensitive learning into the multilabel stream scenario.

## Figures and Tables

**Algorithm 1:**
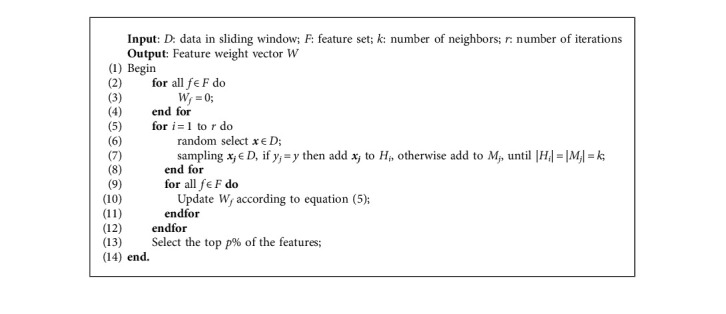
Cost-sensitive ReliefF feature select algorithm.

**Algorithm 2:**
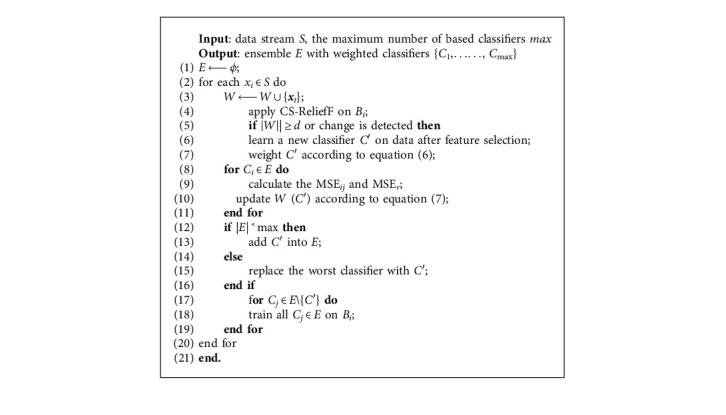
Cost-sensitive based data stream algorithm.

**Table 1:** Description of the datasets.

Data stream	# Inst	# Attrs	# Cls	IR	Drift
HyperPlane	1 m	10	2	5	Gradual
SEA	1 m	3	4	10	Sudden, recurring
LED	1 m	24	10	3	Mixted
Rotating spiral	1 m	40	3	19	None
Spam	9324	500	7	4	Unkown
Sensor	2219803	5	54	54	Unkown
Electricity	45312	10	10	1	Unkown
Airlines	539 383	7	2	2	Unknown

**Table 2:** Average *G*-mean of comparing algorithms.

	VFDT	AUE2	KUE	CSDS
HyperPlane	0.93 ± 0.04 (4)	0.95 ± 0.06 (3)	0.96 ± 0.05 (2)	**0.97** **±** **0.01 (1)**
SEA	0.72 ± 0.10 (4)	0.83 ± 0.12 (3)	0.86 ± 0.22 (2)	**0.88** ± 0.01 **(1)**
LED	0.82 ± 0.02 (4)	**0.87** ± 0.07 **(1)**	0.86 ± 0.06 (2)	0.84 ± 0.02 (3)
Rotating spiral	0.73 ± 0.10 (4)	**0.79** ± 0.05 **(1)**	0.76 ± 0.02 (3)	0.78 ± 0.01 (2)
Spam	0.62 ± 0.08 (4)	0.66 ± 0.02 (3)	0.80 ± 0.04 (2)	**0.86** ± 0.02 **(1)**
Sensor	0.76 ± 0.11 (3)	0.74 ± 0.07 (4)	**0.86** ± 0.06 **(1)**	0.83 ± 0.04 (2)
Electricity	0.60 ± 0.05 (4)	0.64 ± 0.02 (2)	0.62 ± 0.02 (3)	**0.73** ± 0.02 **(1)**
Airlines	0.76 ± 0.07 (3)	0.74 ± 0.08 (4)	**0.83** ± 0.07 **(1)**	0.81 ± 0.03 (2)
Average rank	3.75	2.63	2.00	1.63

**Table 3:** Times of comparing algorithms (seconds).

	VFDT	AUE2	KUE	CSDS
HyperPlane	25.32 (2)	**15.33 (1)**	35.68 (4)	18.10 (3)
SEA	**8.32 (1)**	12.23 (3)	22.07 (4)	10.60 (2)
LED	**29.15 (1)**	36.03 (3)	43.29 (4)	34.65 (2)
Rotating spiral	**9.68 (1)**	16.01 (3)	21.35 (4)	11.04 (2)
Spam	**4.31 (1)**	15.47(4)	14.56(3)	7.45 (2)
Sensor	80.46 (2)	90.24 (3)	104.41 (4)	**66.79 (1)**
Electricity	48.79 (2)	69.41 (3)	77.46 (4)	**31.63 (1)**
Airlines	59.45 (3)	32.23 (1)	60.20 (4)	37.80 (2)
Average rank	1.63	2.63	2.88	1.88

## Data Availability

The datasets and experimental codes can be downloaded from the websites: http://moa.cms.waikato.ac.nz/ and https://github.com/scikit-multiflow/scikit-multiflow.
